# Relationships between Safe Pesticide Practice and Perceived Benefits and Subjective Norm, and the Moderation Role of Information Acquisition: Evidence from 971 Farmers in China

**DOI:** 10.3390/ijerph14090962

**Published:** 2017-08-25

**Authors:** Jianhua Wang, Yuanyuan Deng, Yuting Ma

**Affiliations:** 1 School of Business, Jiangnan University, Lihudadao 1800, Wuxi 214122, China; dyyih04@gmail.com (Y.D.); fyjc1016@163.com (Y.M.); 2 Food Safety Research Base of Jiangsu Province, Jiangnan University, Lihudadao 1800, Wuxi 214122, China

**Keywords:** safe pesticide practice, perceived benefits, subjective norm, policy exposure, market information, China

## Abstract

Improper use of pesticides among farmers has caused food safety issues which are serious threats to public health in China. A central question concerns how to motivate farmers to self-regulate their pesticide usage. The paper aims to identify the influence of an internal driving factor, i.e., perceived benefits, and an external driving factor, i.e., subjective norm, on farmers’ safe pesticide behaviors, and whether the two factors are moderated by the exposure to information on government policies and the market, based on a sample of 971 farmers selected from 5 Chinese provinces. The results revealed that farmers’ safe pesticide usage was predominately driven by perceived benefits whereas external pressure or subjective norm did not play much of a role. Interaction effects were found between the exposure to market information and perceived benefits, and also between subjective norm and exposure to government policy. Extensions agencies are recommended to effectively convey to farmers the benefits to follow safe pesticide practices. Meanwhile, surveillance and monitoring systems should be established so that the prices of their agricultural products are reflected by the quality of the products.

## 1. Introduction

With a tremendous population growth and dramatically increased living standards over the last decades, China has become one of the world’s largest food producers. Central to the growth of food production is the use of modern agricultural technologies including pesticides. China’s pesticides consumption has surged from 500,000 tons in the 1950s to 1.3 million tons in the 2000s, and to the present level of 1.8 million tons in 2015, accounting for one-third of the world’s pesticides and making China the world’s largest pesticide user [1]. Despite its contribution to agricultural production, pesticides have been widely overused by farmers. Chinese farmers sprayed 14 kg/ha pesticides per farming year, which is almost five times higher than farmers from the USA (2.2 kg/ha) and France (2.9 kg/ha) [2].

Pesticide over-use has caused a series of issues related to human health impairment and also to environmental degradation. Pesticide influences public health both directly in terms of 200,000 pesticide-poisoning accidents annually and also indirectly via diet-related diseases due to the presence of pesticide residues in food products [3]. Pesticide residues have been found to have exceeded the maximum residue limits in samples of vegetables and fruits in various Chinese regions including but not limited to Nanjing [4], Xiamen [5] and Shaanxi [6]. Their impacts on environmental pollution and land degradation are also alarming. It was reported that pesticides caused high concentrations of substances that ended up not only in the middle and downstream areas of all major Chinese rivers, including Yangtze, Yellow, Songhua, and Heilong Rivers [7,8] but also in groundwater [9]. In addition, food resources and habitat conditions in aquatic and terrestrial ecosystems have been changed, threatening the livelihood of wildlife [7].

Farmers as direct pesticide users decide how much pesticides are used on their crops. A central question concerns how to motivate farmers to self-regulate pesticide usage and to carry out safe pesticide practices on their own. In our study, we focus on farmers’ compliance of a pre-harvesting interval of a pesticide which refers to the period that must elapse between the last pesticide spray and harvesting of the crop, because following the pre-harvesting interval it is crucial to ensure that pesticide residues in foods are within acceptable limits. A number of studies have investigated the determinants of farmers’ pesticide usage behavior by considering: farmers’ characteristics including education attainment and perception of pesticide technologies [10], training [11,12], risk perception and attitudes towards pesticides [13,14]. Other studies have focused on external driving forces such as governmental regulation [15,16].

However, existing studies have focused on the influence of farmers’ individual characteristics and governmental regulations but have rarely investigated the role of other external factors, i.e., market forces. Nor are the social norm and information acquisition taken into consideration. Because of China’s highly fragmented farming system and strong reliance on administrative power, the market-based mechanism is still being developed so that the quality of agricultural products is not always reflected by their prices, making it crucial to investigate farmer’s marketing behaviors under this transition period in a bid to control the quality of agricultural products. Thus, the analysis of the influence of perceived market revenue of safe pesticide usage on farmers is necessary to identify feasible policy recommendations to ensure proper pesticide usage. Additionally, the investigation from the perspective of external social pressures and information acquisition is necessary to give us insight into the underlying motives which drive farmers’ safe pesticide behaviors. To our knowledge, the interaction between perceived benefits, subjective norm and information acquisition on the enhancement of safe pesticide behaviors has not been studied. Thus, the objective of the paper is to identify the underlying internal factor, i.e., perceived benefits, and external driving factor, i.e., social norm, on farmers’ safe pesticide behaviors and whether these factors are moderated by the exposure to government policies and market information.

The remainder of the paper is organized as follows. Section 2 discusses the conceptual framework and hypothesizes. Section 3 describes the sample characteristics and the measurement of variables. Section 4 discusses the empirical results and Section 5 concludes.

## 2. Conceptual Framework

### 2.1. Perceived Benefits and Safe Pesticide Use Behaviors

Perceived benefit of a technology was found to have determined the acceptance and adoption of the technology. For instance, Iacovou, et al. [17] proposed a framework that incorporates perceived benefit as an important factor for firms to adopt information technologies. Visschers and Siegrist [18] found empirically that perceived benefit was important in explaining the acceptance of energy technologies among Swiss citizens. Stallman and James [19] showed that farmers who believed that they will receive a net benefit were more willing to cooperate with peer farmers to control pests, based on a dataset of 229 Missouri farmers. In our study, perceived benefits refer to farmers’ perception of the usefulness and profitability of agricultural products free from pesticide residues. To the best of our knowledge, no studies have investigated the influence of farmers’ perceived benefits on their compliance with standardized pesticide usage. However, farmers are assumed to evaluate the profitability of different portfolios of pesticides and react dependently using this information. Since standard pesticide spray is a means of loss control and may not necessarily increase yield, it reduces the risk of over-spray and maintains the quality of agricultural products and produce pesticide-free products. Profit-maximizing farmers will not follow these practices if they do not perceive revenues to cover the extra costs. Thus, we hypothesize:

**Hypothesis** **1 (H1).** Farmers who have higher perceived benefits are more likely to practice safe pesticide usage than the farmers with lower perceived benefits.

### 2.2. Subjective Norm and Pesticide Use Behaviors

The theory of planned behavior suggests that normative beliefs result in perceived social pressure or subjective norm to perform that specific behavior [20]. Under the restraint of social norm and social influence promoting certain behaviors, self-ordered individuals comply with these behaviors regardless of whether a law exists to regulate these behaviors [21]. This is because people expect to be recognized as complying with socially desirable behaviors even beyond their interest to gain social acceptance. Based on a sample of 193 Irish conventional drystock farmers, Lapple and Kelley [22] reported that farmers’ decisions to convert to organic farming were significantly related to their perceptions of social pressure from others on carrying out these decisions. We define subjective norm as the pressure from farmers’ relatives, peers, and friends who may push them to follow safe pesticide practices. Due to the fact that inappropriate practices exert strong negative externalities (unsafe food), farmers are expected to follow safe pesticide practices if these practices are shared customary rules of behavior in the local societies. Specifically, we hypothesize:

**Hypothesis** **2 (H2).** Subjective norm or pressure has a positive influence on farmers’ safe pesticide usage.

### 2.3. Information Acquisition and Pesticide Use Behaviors

Limited information explains the lag in the adoption of innovations by farmers [23]. The rationale is that a farmer who expects an economic return will actively gather information which accumulates over time and reach a certain critical level required to adopt a new technology. We consider farmers’ acquisition of two types of information: market information and pesticide-related policy information.

In general, constrained access to market information often dampens farmers’ incentives to use better production techniques such as new varieties or agricultural technologies. For instance, acquisition of market information has increased the price received by farmers and the adoption of improved agricultural seed technology among 800 maize farmers from Uganda [24]. In our study, we define farmer’s acquisition to market information as the richness of sources to obtain market information. With the development of the economy and the rising living standard, the Chinese consumers are paying increasing attention to the quality of food products, shifting from just focusing on food prices. It was reported that Chinese consumers are willing to pay 47% more for vegetables sold with the “Green Food” logo certified by the national government than for conventional vegetables [25]. Thus, we hypothesize:

**Hypothesis** **3 (H3).** Acquisition of market information on safe food products has a positive influence on farmers’ safe pesticide usage.

Following a series of food safety incidents in recent years, the Chinese government has reformed food safety laws, established monitoring and surveillance systems, and strengthened food safety legislative mandates [26]. The goal of these measures was to effectively and timely improve producers’ understanding of the food safety issues and the consequences associated with failing to comply with food safety standards. Farmers’ acquisition of information on governmental policies refers to their understanding of the variety of governmental policies relating to safe food production. Farmers exposed to the above-mentioned information are expected to recognize that they are responsible, and will be held accountable, for pesticide residue problems that may arise from improper use of pesticides. On the other hand, farmers familiar with government’s subsidy policies that reward safe pesticide usage will incentivize them to comply. Thus, we hypothesize:

**Hypothesis** **4 (H4).** Farmers’ acquisition of information on government food safety policies has a positive influence on their safe pesticide usage.

### 2.4. Moderating Role of Information Acquisition

Information-seeking literature conceptualizes individual information seeking behaviors as a process of uncertainty reduction where individuals decide whether to put effort into obtaining information based on the perceived benefits and costs of acquiring that information [27,28]. Since farmers are the direct users of pesticides, they should be made aware of government policies to increase the effectiveness of these policies. The effort for farmers to change their behaviors involves behavioral costs which are not limited to financial costs, but also include the perceived convenience and efforts needed for the specific behavior. Government support and subsidies are useful tools to compensate these behavioral costs. If the target of the policy is to promote better practices, a farmer exposed to these policies will develop better perceived benefits and the social norm may be changed to support these policies as well. Furthermore, following the increasing marketing trend preferring safe agricultural products, farmers’ perceived benefits of safe pesticide usage may be strengthened by a better understanding of market information. In addition to this, we also expect that information acquisition moderates the relationship between subjective norm and safe pesticide behavior. The rationale for this prediction is that information acquisition is positively related to an outcome of subjective norm whereby a positive change in acquiring information should result in a positive change in the subjective norm outcome. For instance, Bamberg [29] observed that subjective norm is a significant predictor of acquiring information about environmentally friendly products. It can be expected that individual farmers acquire information and adapt to social norms formed through the dissemination of information among farmers. Building from these ideas, we examine the impact of the two aforementioned driving factors, i.e., perceived benefits and social norm, arguing that information acquisition, which strengthens the two factors, may moderate this impact. Thus, we predict the following:

**Hypothesis** **5 (H5).** There is a stronger positive relationship between perceived benefits (H5a), social norm (H5b) and behaviors for farmers who have higher exposure to government policies on safe pesticide usage, compared to farmers who are less aware of governmental policies towards safe production.

**Hypothesis** **6 (H6).** There is a stronger positive relationship between perceived benefits (H6a), social norm (H6b) and behaviors among farmers who have a better understanding of market information, compared to farmers who are less informed about the market.

## 3. Methods

### 3.1. Survey

During February–March 2013, a survey was conducted in 5 Chinese provinces following a multi-stage stratified random sampling (a description of the survey has been documented in Wang et al. [14]). First, five major food production provinces from the north (Heilongjiang), middle (Shandong, Henan, and Jiangsu), and south (Zhejiang) regions were selected to account for geographical differences. Next, a random sample of 100 villages was chosen in 20 counties randomly selected in each of the provinces taking into consideration the differentiated county-level economic development. The final stage was to randomly approach 8–12 farmers from each of the 100 villages. University students were recruited and trained as enumerators who carried out the interviews on a face-to-face basis. Among the 986 interviewed farmers, 15 farmers failed to provide information on the core variables and were thus excluded from the analysis, resulting in a sample size of 971.

### 3.2. Sample Characteristics

Sample characteristics are reported in Table 1. It can be observed that males comprised 60.35% of the total sample size, reflecting the fact that pesticide spray was generally done by males; 55.40% of the respondents were aged 45 years or older and a majority of them (78.69%) only received secondary-school or lower education; 67.66% earned household income between 20,000–50,000 Yuan.

### 3.3. Measurements

We measure farmers’ safe pesticide behavior (denoted by *Behavior*) as to whether they follow the pre-harvesting interval of a pesticide, which refers to the period that must elapse between the last pesticide spray and harvesting of the crop. We focused on this particular behavior because following the pre-harvesting interval is crucial and must be followed in order to ensure that pesticide residues on the crop are within acceptable limits. Farmers were asked the extent to which they had followed a pesticide pre-harvesting interval and were scored 1–5 from “not at all” to “extremely often”. Five questions assessed the perceived benefits associated with pesticide residues on foods (*Perceived Benefits* thereafter) by asking whether they think that agricultural products free from pesticide residues will bring about more revenues, better prices, better taste, lowered product costs, and lowered market risks. The responses were scored 1–5 for “not at all” to “very much”. *Subjective Norm* was measured by four questions that asked respondents to indicate whether their decision to produce safe food was affected by the attitudes of their families, friends, peer farmers and government agencies, on a five-point scale ranging from ‘not at all’ to ‘very much’. *Market Information* was measured by three items that asked respondents to rate their exposure to market information on a 5-point scale with 1 = not at all and 5 = very much. *Policy Information* was measured with a 5-item scale that asked the farmers to indicate whether they had known about the local government’s policies including training on pesticide application, propagandas publicizing safe production, standards for safe pesticide application, the penalties and regulations to ensure food safety (1 = Yes and 0 = No). The four control variables considered were: *Age*, *Gender*, *Education Attainment*, and *Farm size*. Definitions and the summary statistics for the variables on *Perceived Benefits*, *Subjective Norm*, *Market Information* and *Policy Information* as well as the dependent variable (*Behavior*) and control variables are given in Table 2.

### 3.4. Data Analysis 

In the first step, the measurement models of the four latent theoretical variables were specified and tested by a confirmatory factor analysis. Following the confirmatory factor analysis, factor scores were created individually for *Perceived Benefits*, *Subjective Norm*, *Market Information*, and *Policy Information*, and used in subsequent analyses. To empirically test what driving factors may influence a farmer’s actual safe pesticide behavior, *Behavior* was regressed on *Perceived Benefits*, *Social Norm* and their interaction effects with *Market Information* and *Policy Information*. Because our dependent variable is an ordinal variable, Ordered Probit models were estimated to identify its determinants. A step-wise estimation procedure was followed to test the robustness of the results. All statistical analyses were performed using Stata (version 13.0; Stata Corp., College Station, TX, USA).

## 4. Results 

### 4.1. Preliminary Analyses

Table 3 presents the results of the confirmatory factor analysis. The factor loadings are satisfactory. All the indicators for *Perceived Benefits*, *Subjective Norm*, and *Policy Information* have factor loadings above 0.40, suggesting that each of the indicators have a satisfactory extent of explanatory power. The only two indicators whose factor loadings are smaller than 0.4 are *Market information 2* (0.15) and *Market information 3* (0.24), but we decided to retain them to ensure an intact structure for *Market Information.*

Table 4 shows an overview of Pearson correlation coefficients among the dependent variable and its influencing factors and control variables. The Behavior score correlated positively and significantly with *Perceived Benefits* (r = 0.25), *Policy Information* (r = 0.13), *Market Information* (r = 0.14), *Age* (r = 0.15), *Gender* (r = −0.07), *Education Attainment* (r = −0.06), and *Farm Size* (r= 0.06). These correlations provide preliminary results to further primary analysis.

### 4.2. Primary Analyses

To test the robustness of the results, the ordered probit models were estimated following a step-wise procedure where Model 1 included only the control variables, Model 2 also included *Perceived Benefits* and *Subjective Norm* in addition to the control variables, and Model 3 also included four interaction terms on top of the independent variables in Model 2. Estimated results are reported in Table 5. To compare the relative model fit among the three models, we conducted log-likelihood ratio tests based on each model’s log-likelihood value. When Model 1 and Model 3 are compared, the likelihood-ratio test statistic equals 103.46, which is larger than the critical value of 20.09 for the 1% significance level (df = 8), suggesting that Model 3 fits significantly better than Model 1. Similarly, Model 3 has a better fit than Model 2 since the likelihood-ratio test statistic equals 23.21, which is larger than critical value of 13.28 (df = 4).

It is shown that *Farm Size* (0.07, *p* < 0.05) and *Age* (0.20, *p* < 0.01) significantly and positively influenced *Behavior*, indicating that older farmers and the farmers operating larger farms were more likely to follow safe pesticide practices. The rationale is that older farmers were more experienced in farming and therefore were more likely to follow safe pesticide practices. Also, our results support the idea that, compared to the counterpart farmers with smaller farms, farmers operating larger farms were more likely to rely on agricultural income than on non-agricultural incomes and were perhaps more concerned about the quality of their products by strictly following safe pesticide practices. In addition, neither *Education Attainment* (0.04, *p* > 0.10) nor *Gender* played a role (−0.12, *p* > 0.10), suggesting that practicing safe pesticide usage did not require much formal education and that female farmers did not show a higher likelihood to follow safe practices than male farmers.

Model 2 shows that *Perceived Benefits* had a significant and positive impact (0.13, *p* < 0.01) on *Behavior*, supporting hypothesis H1. However, H2 was rejected because *Subjective Norm* has an insignificant coefficient of −0.01 (*p* > 0.10). Both *Market Information* (0.33, *p* < 0.01) and *Policy Information* (0.27, *p* < 0.01) had significant influences on *Behavior*, confirming hypotheses H3 and H4, respectively.

Model 3 revealed that the main effects shown in Model 2 still hold, although with slightly different coefficients. Further, it showed that the interaction between *Perceived Benefits* and *Policy Information* had an insignificant impact (0.05, *p* > 0.10), rejecting hypothesis H5a. With regard to hypothesis H5b and H6a, interaction effects were confirmed with a positive and significant coefficient for *Perceived Benefits* × *Market Information* (0.20, *p* < 0.05) and between *Subjective Norm* and *Policy Information* (0.20, *p* < 0.01). Thus, both hypotheses H5b and H6a are supported by these findings. Finally, there is no support for hypothesis H6b where no interaction effect was evident for *Subjective Norm* × *Market Information* (0.01, *p* > 0.10). Notably, *Age* had a positive and significant influence on *Behavior* in all three models whereas *Farm size* had significant impacts on *Behavior* in Models 2 and 3 but not in Model 1. The influence of *Education Attainment* was insignificant, regardless of the models.

## 5. Discussion

Food safety issues are causing major public health issues in China. The agricultural production process provides opportunities for contamination from farmers’ improper use of agrochemicals, especially pesticides, leading to the presence of unsafe pesticide residues in food products. Therefore, understanding the motives behind farmers’ safe pesticide practices is crucial. The main objective of the paper is to identify the underlying internal and external driving factors of farmers’ safe pesticide behaviors, i.e., perceived benefits and subjective norm, and whether these motivations are moderated by exposure to government policies and market information. 

First, farmers’ perceived benefits of the safe practices that play a dominant role whereas external pressure or subjective norm does not play much of a role. That means that farmers perform safe pesticide practices because they care about farming revenues, suggesting some evidence of intrinsic motivation. Further, it was reported that the influence of perceived benefits is further facilitated by market information. Policy recommendations arising from this result is that attention could be paid by extensions agencies to effectively convey to farmers the various benefits associated with safe pesticide practices. Meanwhile, surveillance and monitoring systems could be strengthened so that the quality of their agricultural products is reflected by the quality of these products. Due to the highly fragmented farmland, China’s ability to monitoring the quality of individual farms is impeded. However, it is practical to monitor food products sold in stores and supermarkets, which in turn may ultimately put pressure on farmers. This could also be coupled with a market-based pricing system where food products free from pesticide residues could have higher prices. This strategy has great potential under the context where the Chinese consumers are willing to pay a substantially higher price for safe food products.

Second, we found that subjective norm does not function alone in enhancing safe pesticide behaviors, but it was found as being influential when it is coupled with policy exposure. This is probably because farmers live in rural environments where a large majority of residents generally have low environmental awareness. Therefore, in the context of China’s rural context, enhancing subjective norms alone that regulate farmers’ behaviors will be in vain. However, increasing policy exposure to rural residents, in general, will enhance social norms preferring safe pesticide usage, which in turn bolster farmers’ behavioral change. 

## 6. Conclusions

In summary, this study shows that farmers performed safe pesticide practices because they cared about the possible farming revenues arising from safe food products they produce, rather than because of external pressure or subjective norm. Thus, our findings suggest that extensions agencies could effectively convey to farmers the various benefits associated with safe pesticide practices. Moreover, the influence of perceived benefits is further facilitated by market information. This result suggests that surveillance and monitoring systems could be strengthened so that the quality of their agricultural products is reflected by the quality of these products.

## Figures and Tables

**Table 1:** Description of the samples.

Characteristics	Categories	N	%	Characteristics	Categories	N	%
*Age (years)*	<18	8	0.82	*Education Attainment*	Primary school or less	283	29.15
	18–25	75	7.72		Secondary school	481	49.54
	26–45	350	36.05		High school	152	15.65
	45–60	403	41.50		Vacational college	26	2.68
	60	135	13.90		Bachelor or higher	29	2.99
*Family size (persons)*	*1–2*	*68*	*7.00*	*Household income*	<20,000 Yuan	117	12.05
	3	244	25.13		20,000–30,000 Yuan	322	33.16
	4	334	34.40		30,000–50,000 Yuan	335	34.50
	>5	325	33.47		50,000–100,000 Yuan	196	20.19
*Gender*	Male	586	60.35		>100,000 Yuan	1	0.10
	Female	385	39.65				

Notes: 6.25 Yuan ≈ $1 (2013 data).

**Table 2:** Variable descriptions and summary statistics.

Variables	Description	Mean	S.D.	N
Behavior	To what extent do you follow a pesticide’s pre-harvesting interval?	3.65	1.14	971
Perceived benefit 1	Do you think that agricultural products free from pesticide residues will bring about more revenues?	3.79	0.85	971
Perceived benefit 2	Do you think that agricultural products free from pesticide residues will bring about better prices?	3.83	0.70	971
Perceived benefit 3	Do you think that agricultural products free from pesticide residues will lower production costs?	3.61	0.74	971
Perceived benefit 4	Do you think that agricultural products free from pesticide residues will have a better taste?	3.83	0.71	971
Perceived benefit 5	Do you think that agricultural products free from pesticide residues will lower market risk?	3.73	0.66	971
Subjective norm 1	Do the attitudes of your families affect your decision to produce safe food?”	3.28	0.99	971
Subjective norm 2	Do the attitudes of your friends affect your decision to produce safe food?”	2.92	0.99	971
Subjective norm 3	Do the attitudes of your peer farmers affect your decision to produce safe food?”	3.12	0.91	971
Subjective norm 4	Do the attitudes of the government agencies affect your decision to produce safe food?”	3.29	1.09	971
Market information 1	How do you rate your accessibility to market information?	2.18	0.75	971
Market information 2	Could you obtain market information from the village council?	0.22	0.41	971
Market information 3	Could you obtain market information from the farmers’ cooperatives?	0.11	0.32	971
Policy information 1	Do you know about the local government’s training on pesticide application?	0.30	0.46	971
Policy information 2	Do you know about the local government’s propagandas publicizing safe production?	0.47	0.50	971
Policy information 3	Do you know about the local government’s penalty for farmers who violate safe pesticide use?	0.43	0.50	971
Policy information 4	Do you know about the local government’s established standards for safe pesticide application?	0.37	0.48	971
Policy information 5	Do you know about the local government’s released regulatory documents to ensure food safety?	0.39	0.49	971
**Control variables**				
Gender	Scored 1 if a farmer is female, otherwise 0	0.40	0.49	971
Age	Scored 1 if a farmer is younger than 18 years old; 2 if 19–25 years old; 3 if 26–45 years old; 4 if 46–60 years old; and 5 if older than 60 years old.	3.60	0.85	971
Education Attainment	Scored 1 if a farmer received primary school education or lower; 2 if middle school; 3 if high school; 4 if vocational college; and 5 if bachelor or higher	2.01	0.91	971
Farm Size	Scored 1 if farm size is less than 2 mu; 2 if 2–3 mu; 3 if 3–6 mu; and 5 if more than 6 mu	2.61	1.07	971

Notes: 15 mu = 1 hectare.

**Table 3:** Standardized factor-loadings for the observed indicators.

Variables	Perceived Benefits	Subjective Norm	Market Information	Policy Information
Perceived benefit 1	0.42			
Perceived benefit 2	0.53			
Perceived benefit 3	0.63			
Perceived benefit 4	0.76			
Perceived benefit 5	0.55			
Subjective norm 1		0.82		
Subjective norm 2		0.75		
Subjective norm 3		0.59		
Subjectivenorm 4		0.40		
Market information 1			0.58	
Market information 2			0.15	
Market information 3			0.24	
Policy information 1				0.67
Policy information 1				0.41
Policy information 2				0.53
Policy information 3				0.77
Policy information 4				0.76

**Table 4:** Spearman rank correlation coefficients among the variables (n = 971).

Variables	1	2	3	4	5	6	7	8	9
1 Behavior	1.00								
2 Subjective Norm	0.01	1.00							
3 Policy Information	0.13 ***	0.16 ***	1.00						
4 Market Information	0.14 ***	−0.03	0.23 ***	1.00					
5 Perceived Benefits	0.25 ***	0.10 ***	0.10 ***	0.04	1.00				
6 Gender	−0.07 **	−0.04	0.05	−0.02	−0.01	1.00			
7 Age	0.15 ***	−0.08 **	0.02	−0.01	0.06 *	−0.08 ***	1.00		
8 Education	−0.06 *	0.12 ***	0.05	0.10 ***	0.04	0.04	−0.47 ***	1.00	
9 Farm Size	0.06 *	0.05 *	0.07 **	−0.01	−0.03	−0.15 ***	−0.02	−0.13 ***	1.00

Notes: * *p* < 0.1; ** *p* < 0.05; *** *p* < 0.01.

**Table 5:** Results of the step-wise Ordered Probit model.

Independent Variables	Model 1	Model 2	Model 3
Control variables			
*Gender*	−0.12 (0.07)	−0.13 * (0.07)	−0.14 ** (0.07)
*Age*	0.20 *** (0.05)	0.16 *** (0.05)	0.15 *** (0.05)
*Education Attainment*	0.04 (0.04)	−0.01 (0.04)	0.00 (0.04)
*Farm Size*	0.07 ** (0.03)	0.07 ** (0.03)	0.06 (0.03)
Main effects			
*Perceived Benefits* (H1)		0.13 *** (0.05)	0.11 ** (0.05)
*Social Norm* (H2)		−0.01 (0.04)	0.00 (0.04)
*Market Information* (H3)		0.33 *** (0.05)	0.35 *** (0.05)
*Policy Information* (H4)		0.27 *** (0.08)	0.29 *** (0.08)
Interaction effects			
*Perceived Benefits* × *Policy Information* (H5a)			0.05 (0.05)
*Subjective Norm* × *Policy Information* (H5b)			0.20 *** (0.06)
*Perceived Benefits* × *Market Information* (H6a)			0.20 ** (0.09)
*Subjective Norm* × *Market Information* (H6b)			0.01 (0.09)
Log-likelihood	−1403.31	−1361.90	−1351.58
N	971	971	971

Notes: * *p* < 0.1; ** *p* < 0.05; *** *p* < 0.01.
